# Supplementary material for: Detection of Hepatitis E Virus Genotype 3 in Feces of Capybaras (Hydrochoeris hydrochaeris) in Brazil
Source: Viruses. 2023 Jan 24;15(2):335. doi: 10.3390/v15020335 (PMC9959927; doi:10.3390/v15020335)
Supplement: Supplementary file 1 [file viruses-15-00335-s001.zip › Figure S3B.pdf]

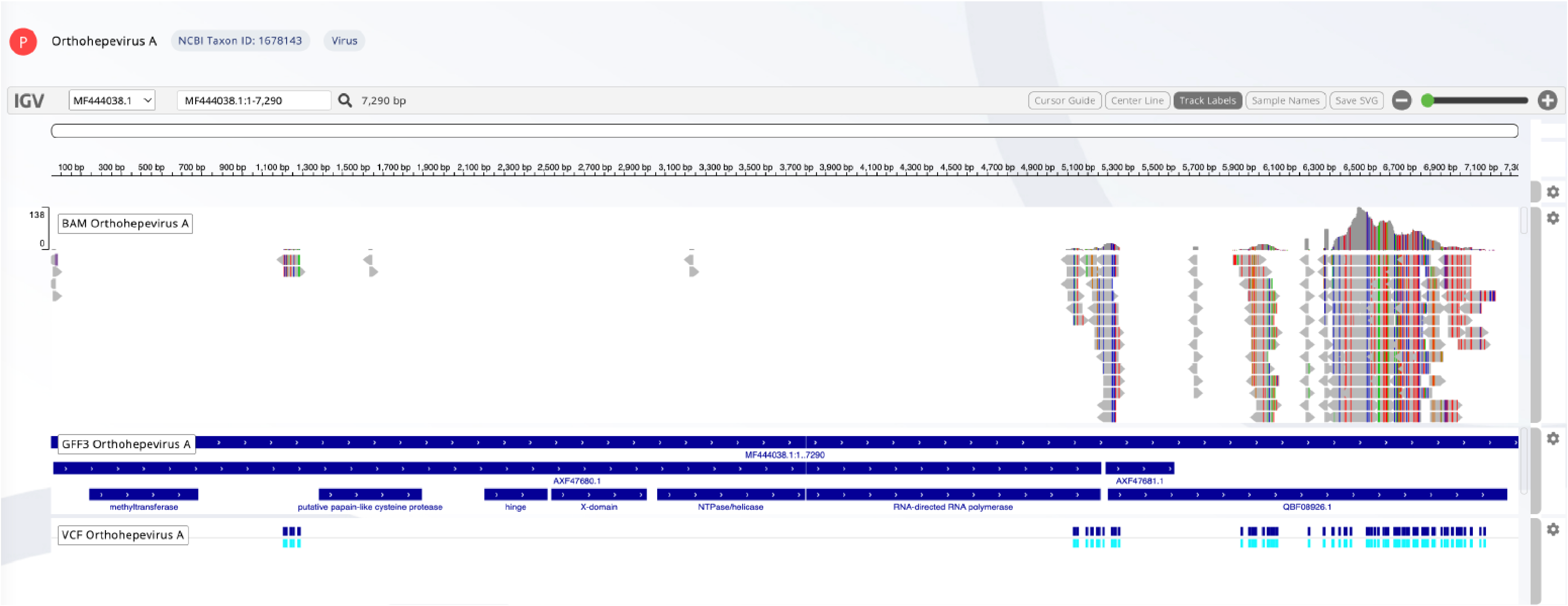

Figure S3B. Mapping coverage plot in the HEV reference genome according to the Varsmetagen online platform (https://varsomics.com/varsmetagen/).
