# Supplementary material for: Detection of Hepatitis E Virus Genotype 3 in Feces of Capybaras (Hydrochoeris hydrochaeris) in Brazil
Source: Viruses. 2023 Jan 24;15(2):335. doi: 10.3390/v15020335 (PMC9959927; doi:10.3390/v15020335)
Supplement: Supplementary file 1 [file viruses-15-00335-s001.zip › Figure S1.pdf]

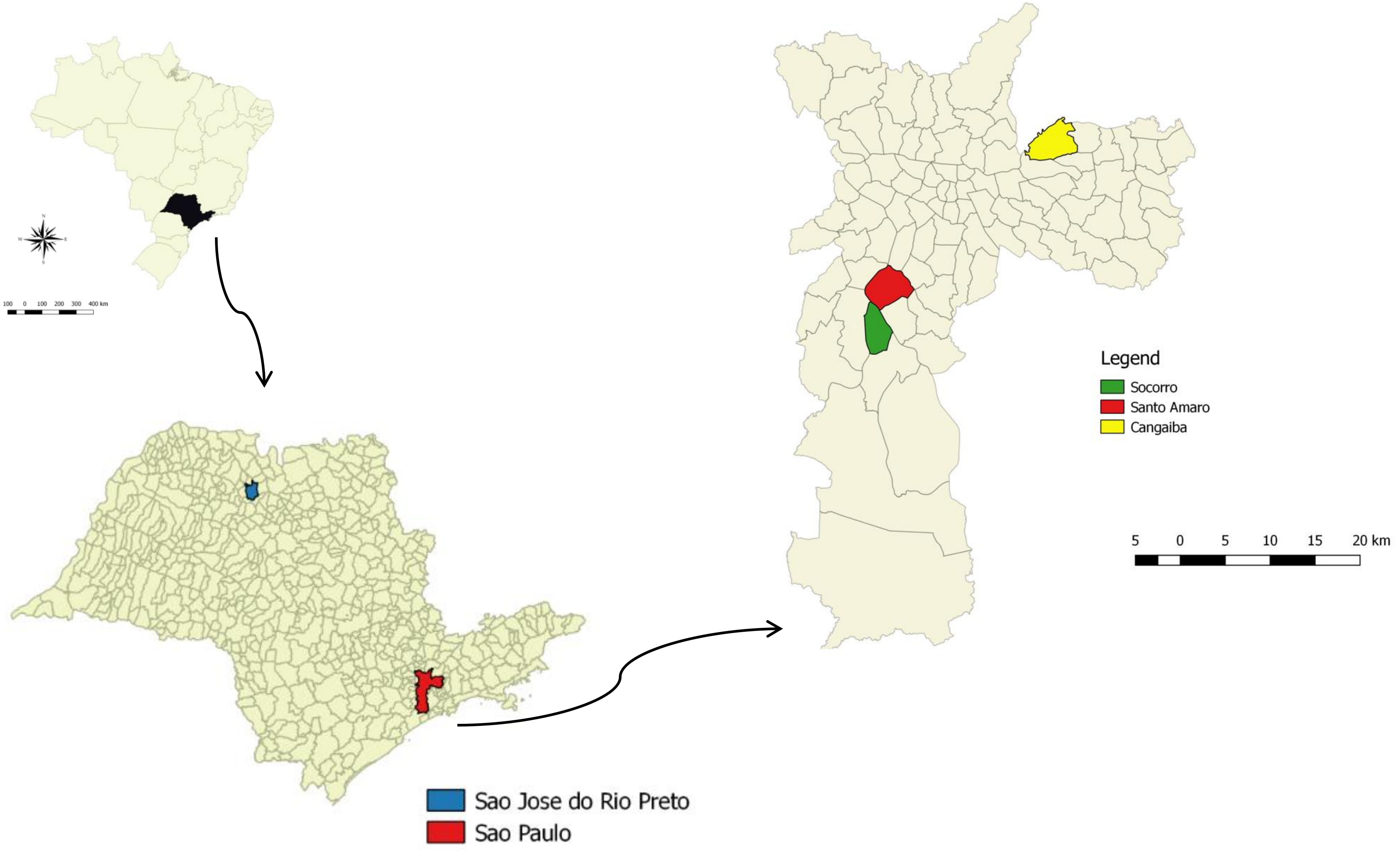

Figure S1. Map of São Paulo state, Southwestern region, Brazil; highlighting municipalities (in red and blue) and São Paulo municipalities' districts (in red, green and yellow) from which samples were collected from free-living capybaras in urban parks between 2018 and 2020. Up Left: Map of Brazil stressing São Paulo state. Down: Municipalities surveyed: São Paulo and São José do Rio Preto. Up Right: Districts surveyed at São Paulo municipality. Map was generated with QGIS software v2.14.9 (https://www.qgis.org/pt_BR/site/about/index.html).
